# Developing a theoretical relationship between electrical resistivity, temperature, and film thickness for conductors

**DOI:** 10.1186/1556-276X-6-636

**Published:** 2011-12-22

**Authors:** Fred Lacy

**Affiliations:** 1 Electrical Engineering Department, Southern University and A&M College, Pinchback Hall, Rm 428, Baton Rouge, LA, 70813, USA

**Keywords:** Callendar-van Dusen, conductivity, mean free path, nanofilm, resistance temperature detector, temperature sensor, thin film

## Abstract

**Abstract:**

Experimental evidence has made it clear that the size of an object can have an effect on its properties. The electrical resistivity of a thin film will become larger as the thickness of that film decreases in size. Furthermore, the electrical resistivity will also increase as the temperature increases. To help understand these relationships, a model is presented, and equations are obtained to help understand the mechanisms responsible for these properties and to give insight into the underlying physics between these parameters. Comparisons are made between experimental data and values generated from the theoretical equations derived from the model. All of this analysis provides validation for the theoretical model. Therefore, since the model is accurate, it provides insight into the underlying physics that relates electrical resistivity to temperature and film thickness.

**PACS:**

73.61.At; 73.50.Bk; 72.15.Eb; 72.10.d; 63.20.kd.

## Introduction

Nanotechnology is an emerging branch of science that seeks to understand how materials operate and function when at least one of their dimensions is less than 100 nm in size. Through various experimental studies, it is understood that when materials shrink to dimensions on the nanoscale, many of the properties or characteristics that they display in bulk form are no longer valid [1-5]. Mechanical, thermodynamic, electrical, and optical properties have been shown to be altered because of the size difference. The reasons for this change in properties are due to increased surface interactions as well as absorption and scattering effects [1-5].

Several studies have shown that diminishing one of the dimensions of a conductor will alter the electrical resistivity of the material [6-22]. The electrical resistivity that the material has when it is in bulk form is not the resistivity that the material has when it is nanosized. It is understood that this change occurs because the mean free path of conduction electrons is reduced due to increased scattering effects. Obviously, the electrical resistivity, and other properties, of thin films may behave differently than expected if the thickness of the material becomes sufficiently small.

Numerous research studies have developed or used theoretical models to characterize and explain the behavior of the electrical resistivity of metallic thin films as a function of film thickness [6-22]. Each of these models is less than ideal for at least one of the following reasons. The model does not account for enough of the different scattering effects to be practical [6-11,13,18]. The equation produced from the model is very complex and/or does not have a closed form solution [6,7,9,10,12-15,18,20,21]. When a complex equation is reduced to a simpler one using various assumptions, the result is an equation that is inaccurate and/or not very practical [8,11-13,16-18]. The model and/or equation is too simple and does not explain a key aspect of the underlying physics [8,19,21]. When compared with the experimental data, the equation does not provide a good fit, and therefore, the model and/or equation is not accurate [6,7,11,13,15,18,22]. Because of the aforementioned shortcomings, a new model explaining the resistivity and film thickness relationship is needed.

Films of platinum and nickel have been used successfully to sense or measure temperatures based on changes in the electrical resistivity of these materials. These devices are known as resistance temperature detectors, and they have a well-established and highly repeatable resistance-temperature relationship that increases linearly as temperature increases [23,24]. Furthermore, a theoretical model has recently been created to explain the mechanisms that are responsible for the resistivity-temperature relationship (F Lacy, unpublished work) [25]. However, this model was created for bulk materials and not for nanoscale-dimensioned materials. Thus, modifications to the model and/or another model are needed in order to elucidate the physical mechanisms behind the resistivity-temperature-film thickness relationship for conductors.

To help explain the behavior of nanosized conductors, a two-dimensional theoretical model was created and analyzed such that the relationship between the electrical resistivity, temperature, and film thickness could be understood. The result from this analysis is an equation which was plotted to show that it provides a good match with experimental results. Based on the comparisons with experimental findings, the theoretical model provides reasonable results and thus offers insight into the underlying physics of the interaction between electrons, scattering objects, and phonons for nanoscale conductors.

### Resistivity-film thickness model

#### Surface scattering effects

**Figure 1:**
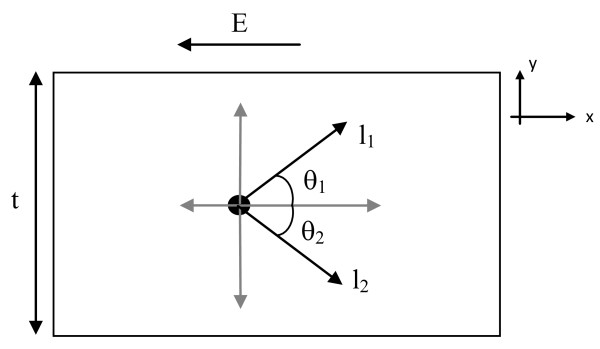
**Two-dimensional structure with an electron that will not be scattered by the surface**. The structure contains an electron in the presence of an electric field when the electron will not be scattered by the surface.

**Figure 2:**
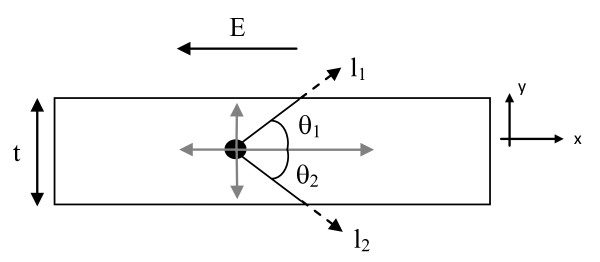
**Two-dimensional structure with an electron that will be scattered by the surface**. The structure contains an electron in the presence of an electric field when the electron will be scattered by the surface.

In order to obtain a relationship between the electrical resistivity and film thickness, the physical models shown in Figures 1 and 2 will be used. The thin film conductor will have a thickness, *t*, and it is assumed to have smooth or even surfaces. To simplify this analysis, only the interaction between the electron and the boundary of the conductor will be considered in the analysis. In other words, no electron atomic interactions will be directly considered for this model, but these interactions are assumed and will lead to electrons traveling an average distance, *l*. The conduction electron will travel a distance of *l *(which is known as the mean free path) unless it is scattered by the surface of the material. When the aforementioned interaction occurs, the electron will travel a distance less than the mean free path (this shorter distance will be determined in this section).

To further simplify the analysis, the electron will be located at the 'average' position in the *y *direction, and thus, it will be placed equidistant from the top and bottom of the material (i.e., the electron will be located in the center of the material). The model is divided into quadrants, and because of the symmetry, only the first quadrant needs to be considered or analyzed. Based on this model, two different scenarios exist.

The first situation occurs when the bulk mean free path of the electrons is less than *t*/2 (as shown in Figure 1); mathematically, *l*_1 _= *l*_2 _= *l*_bulk _≤ *t*/2. As a result, based on this model and the symmetry in this model, the mean free path of the average conduction electron will not be altered or the electron will not be scattered by the surface. In general, *l*_film _= *βl*_bulk _where *l*_film _is the mean free path for conduction electrons in a thin film of thickness *t*, *l*_bulk _is the bulk mean free path, and *β *is the ratio between the two terms due to scattering. When *l*_1 _= *l*_2 _= *l*_bulk _≤ *t*/2 and when there is no surface scattering, this proportionality constant will be equal to 1, or the mean free path for the film will not be different than its bulk counterpart, and thus

(1) $$\rho =\rho_{0},$$

where *ρ*_0 _is the bulk resistivity of the material.

The second case occurs when the mean free path of the electrons is greater than *t*/2 (as shown in Figure 2); mathematically, *l*_1 _= *l*_2 _= *l*_bulk _≥ *t*/2. When this happens, the electron will occasionally be scattered by the surface. Based on this scenario, the ratio of the thin film and bulk mean free paths is given by

(2) $$\beta =\frac{\frac{2}{\pi }\left[\int_{0}^{\sin^{-1}\frac{t/)2}{l_{\text{bulk}}}}l_{\text{bulk}}\cos\theta d\theta +\int_{\sin^{-1}\frac{t/)2}{l_{\text{bulk}}}}^{\pi /)2}\frac{t/)2}{\tan\theta }d\theta \right]}{\frac{2}{\pi }\int_{0}^{\pi /)2}l_{\text{bulk}}\cos\theta d\theta },$$

where the electron is not scattered by the surface in the first integral in the numerator, and the electron is scattered by the surface in the second integral in the numerator. It is known that $\int \frac{1}{\tan\theta }d\theta =\int \cot\theta d\theta =\ln\left|\sin\theta \right|$, therefore, Equation 2 becomes

(3) $$\beta =\frac{\left[{\left[l_{\text{bulk}}\sin\theta \right]}_{0}^{\sin^{-1}\frac{t/)2}{l_{\text{bulk}}}}+\frac{t}{2}{\left[\ln\left|\sin\theta \right|\right]}_{\sin^{-1}\frac{t/)2}{l_{\text{bulk}}}}^{\pi /)2}\right]}{{\left[l_{\text{bulk}}\sin\theta \right]}_{0}^{\pi /)2}}.$$

After evaluating Equation 3, the ratio for the thin film mean free path to the bulk mean free path is

(4) $$\beta =\frac{\frac{t}{2}\left[1-\ln\frac{t/)2}{l_{\text{bulk}}}\right]}{l_{\text{bulk}}}.$$

Now, by defining $\kappa =\frac{t∕2}{l_{\text{bulk}}},$ where *κ *is a constant such that 0 < κ ≤ 1,

(5) $$\beta =\kappa \left[1-\ln\kappa \right],$$

where, again, *β *is the mean free path ratio of thin film and bulk materials (when *l*_1 _= *l*_2 _= *l*_bulk _≥ *t*/2) or equivalently *β *= *l*_film_/*l*_bulk_. It is seen that when *κ *= 1, then *β *will be equal to 1 and *l*_film _= *l*_bulk _as expected.

The electrical resistivity of thin films can be found from the equation $\rho =\frac{m}{ne^{\text{2}}\tau_{\text{avg}}}$, where *τ*_avg _is the average scatter time and is related to the mean free path by equation *l *= v_F_*τ_avg_, where *v_F _*is the Fermi velocity. Thus, the resistivity can also be written as $\rho =\frac{mv_{F}}{ne^{2}l}$. Therefore, the electrical resistivity as a function of film thickness can be expressed as

(6) $$\rho =\frac{\rho_{0}}{\kappa \left[1-\ln\kappa \right]},$$

where $\rho_{0}=\frac{mv_{F}}{ne^{2}l_{\text{bulk}}}$ and represents the bulk resistivity of the material.

#### Additional scattering effects

In addition to conduction electrons being scattered by the surface of the material, several other scattering mechanisms exist in the material to alter the path of these electrons. The most significant of these mechanisms is scattering from grain boundaries, scattering from uneven or rough surfaces, and scattering due to impurities. These effects are dependent on the procedures and conditions used to fabricate the thin films, and thus, it is very difficult to quantify each of these effects without measurement. However, what is clear about these additional scattering mechanisms is that processing techniques and impurity concentration will have a larger effect on the bulk resistivity and that grain boundary size and rough surface scattering are more prominent for smaller film thicknesses.

The end result of these additional scattering effects is a further reduction in the mean free path of the conduction electron (and thus an increase in the electrical resistivity). Since these additional scattering effects may affect experimental measurements, the results from the resistivity-film thickness model will be enhanced to make it more adaptive and capable of producing data that are compatible with the experimental results.

If the film has a thickness larger than two times the mean free path and a measured reference resistivity larger than *ρ*_0_, then the fabrication or processing technique as well as impurities in the material have increased the resistivity of the film. As a result, the resistivity term in Equation 1 has to be modified. A scaling factor is used to modify the bulk resistivity such that

(7) $$\rho =\rho_{0}^{\prime }=c\rho_{0},$$

where *ρ*_0 _is the bulk resistivity for the material, *c *is a constant (*c *≥ 1), and $\rho_{0}^{\prime }$ is the modified bulk resistivity due to the additional scattering effects. Again, Equation 7 is true when *t *≥ 2*l*_bulk_.

Likewise, if the film has a thickness smaller than two times the mean free path and a measured reference resistivity larger than *ρ*_0_, then again, the fabrication technique and material impurities have increased the resistivity of the film. As a result, the bulk resistivity term in Equation 6 has to be modified. Similar to Equation 7, the bulk resistivity is modified such that $\rho_{0}^{\prime }=c\rho_{0}$. Also, scattering from grain boundaries and rough surfaces can have a significant or dramatic effect when the film thickness is very small (< 20 nm). Therefore, an 'effective film thickness' can be used to account for this result. Thus, this effective thickness is given as *t*' = *t *- *η *where *t *is the film thickness, *η *is the reduction factor, and *t*' is the effective thickness. As a result, *κ *which equals $\frac{t∕2}{l_{\text{bulk}}}$ or $\frac{t}{2l_{\text{bulk}}}$ will be modified and can be written as $\kappa^{\prime }=\frac{t-\eta }{2l_{\text{bulk}}}$. After these adjustments have been made, the resistivity can be written as

(8) $$\rho =\rho_{0}^{\prime }=\frac{c\rho_{0}}{\kappa^{\prime }\left[1-\ln\kappa^{\prime }\right]},$$

and this represents the thin film resistivity with all scattering effects taken into account when *t *≤ 2*l*_bulk_

### Resistivity-temperature model for thin films

A model has recently been developed that relates electrical resistivity to temperature (F Lacy, unpublished work) [25]. The primary equation from this model is given as

(9) $$\rho =\rho_{0}\left[\frac{1}{\frac{\left[2\sqrt[{\delta }]{\gamma /)kT}-b\right]\left(\tau_{1}/)\tau_{2}-1\right)}{2a+b}+1}\right],$$

where $\rho_{0}=\frac{m}{ne^{2}\tau_{2}}$, *a *is the atomic radius, *b *is the size of the opening between atoms (when the atoms are stationary), *τ*_1_/*τ*_2 _is the ratio of travel time before scattering when an electron is in the gap to when an electron is not in a gap, *δ *is 1, *k *is Boltzmann's constant, *T *is temperature (in kelvin), and *γ *is the proportionality term in the energy equation $U=\frac{\gamma }{r^{\delta }}$.

Additionally, it has recently been shown that thin films of platinum exhibit an electrical resistance (and thus resistivity) that increases as the temperature increases, but it is not strictly linear as bulk platinum would be [26-28]. These films had thicknesses that were nanometer in size, and thus, it is believed that this nonlinear effect is the result of the size of these nanoscale conductors. Equation 9 was derived for bulk materials, and it represents the resistivity as a function of temperature for bulk materials. However, by appropriately altering Equation 9, this equation can effectively represent a conductor of nanometer thickness.

Again, when materials become smaller in size, their properties are altered. As a relevant example, melting point depression is a phenomenon that occurs in which the melting point of a material becomes lower when the size of that material is reduced [29-31]. Because the properties of molecules are altered when the material becomes sufficiently small, these molecules gain enough energy at lower temperatures to change from the solid to the liquid state.

Likewise, it would be reasonable to expect that the electrical resistivity property of a conductor would behave like the melting property since energy of the atoms will affect both properties. It would be logical to expect that the electrical resistivity at lower temperatures would be similar for bulk and nanofilm conductors, whereas at higher temperatures, a difference would emerge. This phenomenon has been demonstrated experimentally [26-28], so modifications to Equation 9 will aid in understanding why this phenomenon occurs.

Equation 9 contains a term, *γ*, that is the proportionality variable relating the energy of the atoms to the separation distance between atoms. This parameter was initially selected to be constant, and this led to a good match with the Callendar-van Dusen coefficients [25]. After performing additional analysis, it was determined that by varying *γ*, a better match for the Callendar-van Dusen coefficients was obtained (F Lacy, unpublished work). As a result of this aforementioned analysis and since *γ *is a term that is related to the energy of the atoms, it is reasonable that further varying this term will have the desired effect of altering the resistivity from its bulk values to its nanoscale values.

### Resistivity-film thickness-temperature equation

The equation that relates the electrical resistivity to film thickness and the equation that relates electrical resistivity to temperature can be combined to produce a composite equation. This composite equation is

(10) $$\rho =c\rho_{0}\left[\frac{1}{\frac{\left[2\sqrt[{\delta }]{\gamma /)kT}-b\right]\left(\tau_{1}/)\tau_{2}-1\right)}{2a+b}+1}\right]\;\text{if}\;t\geq 2l_{\text{bulk}}$$

or

(11) $$\rho =\frac{c\rho_{0}}{\kappa^{\prime }\left[1-\ln\kappa^{\prime }\right]}\left[\frac{1}{\frac{\left[2\sqrt[{\delta }]{\gamma /)kT}-b\right]\left(\tau_{1}/)\tau_{2}-1\right)}{2a+b}+1}\right]\;\text{if}\;t\leq 2l_{\text{bulk}},$$

where the particular equation used depends upon the relationship between the film thickness (*t*) and the mean free path of the conduction electrons in that material (*l*_bulk_). Thus, after a film has been manufactured at a particular thickness, the response of that film can be completely characterized by Equation 10 or 11, depending upon the thickness of that film.

## Results

Having developed the theoretical model, the corresponding theoretical equations can be validated by generating data from the appropriate equation and matching these data to experimental results. A good fit between the two sets of data will confirm the accuracy of the theoretical model. It is noted that experimental data are available for resistivity vs. film thickness as well as resistivity vs. temperature. Thus, for the resistivity vs. film thickness experimental data, Equation 11 can be validated by fixing the temperature to room temperature value (as a result of this, Equation 11 reduces to Equation 8). The same applies for Equations 7 and 10. For the resistivity vs. temperature experimental data, Equation 11 can be validated by fixing the film thickness (as a result of this, Equation 11 reduces to Equation 9). Thus, validating Equation 8 using experimental resistivity vs. film thickness data and validating Equation 9 using experimental resistivity vs. temperature data will automatically validate Equation 11 which is the composite of Equations 8 and 9. Again, the same applies for Equations 7, 9, and 10.

The electrical resistivity for metals can be plotted as a function of film thickness using Equation 8. Using experimental data from other researchers, Equation 8 can be viewed as a curve fit with *c *and *κ*' (or equivalently *η*) as adjustable parameters. Again, a good match between experimental and theoretical data will demonstrate that the theoretical model is reliable and valid.

The parameters for the theoretical model that are needed to generate data (and thus compare with experimental data) are provided in Table 1. As can be seen from these data, these parameters include values for *c *that range from 6.55 to 1.00. This means that the modified bulk resistivities range from values that are 6.55 times larger than the bulk resistivity to values that exactly match the bulk resistivity. Likewise, values for *η *range from 59.5 to 0.00 nm. This means that the effective film thicknesses range from 59.5 nm smaller than the actual or measured thickness to values that exactly equal the measured thickness.

**Table 1:** Parameters used to generate the theoretical resistivity-film thickness data

**Figure**	***c***	***η *(nm)**
3	1.00	11.40
4a	2.17	1.50
4b	1.40	1.50
5	1.06	59.50
6a	4.61	0.00
6b	6.55	2.45
6c	3.07	5.50

**Figure 3:**
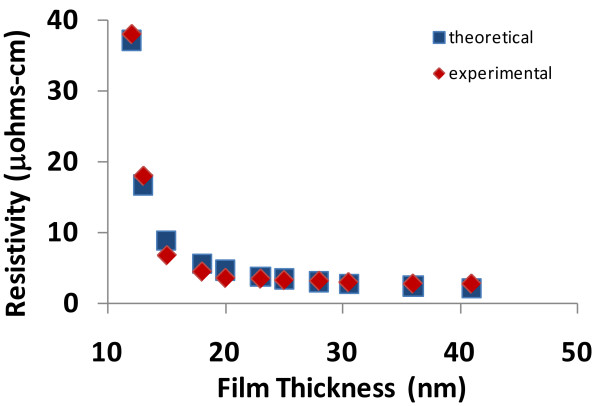
**Comparison of theoretical and experimental resistivities for copper as a function of film thickness**. (The experimental data is adapted from Liu et al. [11]).

**Figure 4:**
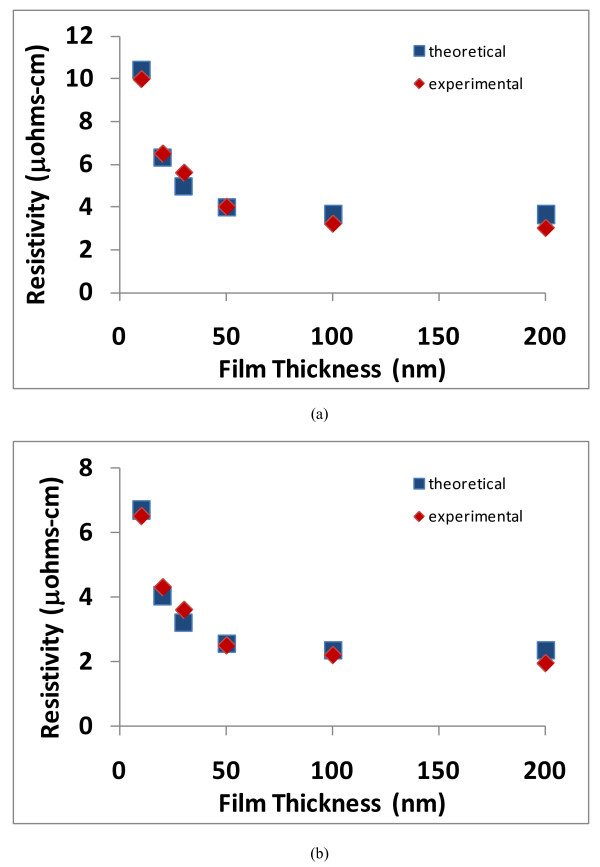
**Comparison of theoretical and experimental resistivities for copper as a function of film thickness**. The graphs in (**a**) and (**b**) represent copper films using different fabrication parameters (the experimental data is adapted from Rossnagel and Kuan [12]).

**Figure 5:**
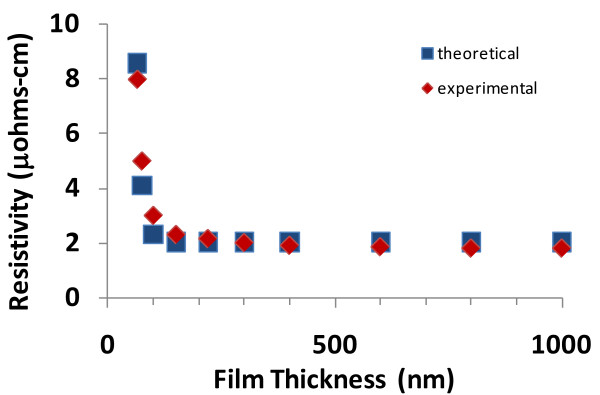
**Comparison of theoretical and experimental resistivities for silver as a function of film thickness**. (The experimental data is adapted from Dayal et al. [13].)

**Figure 6:**
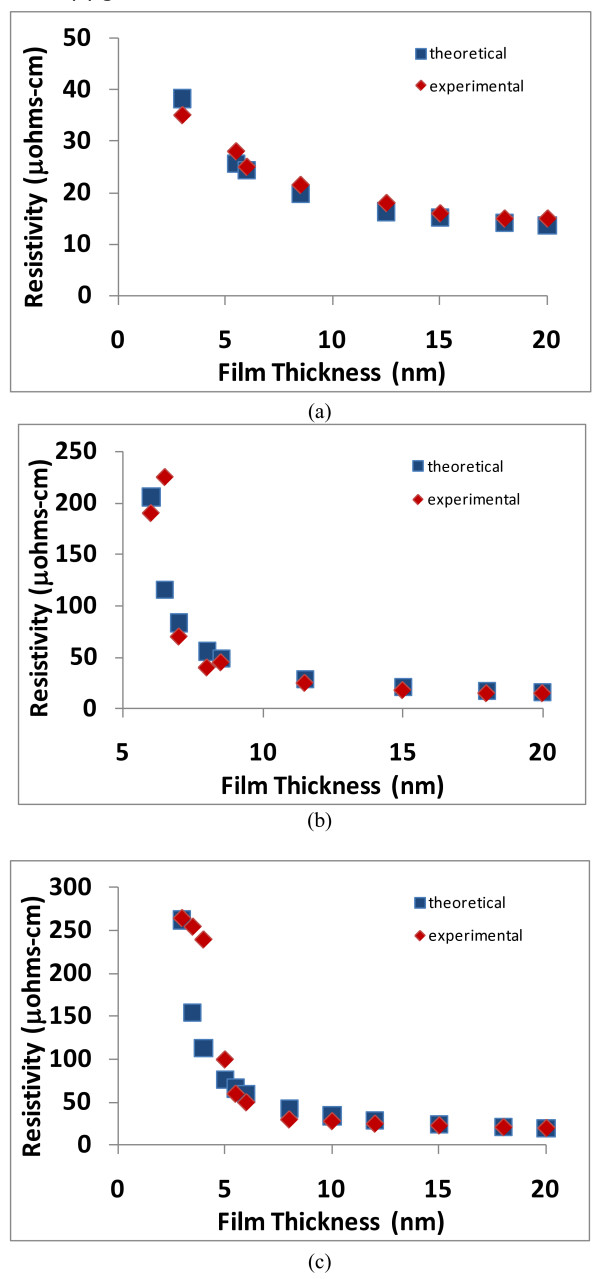
**Comparison of theoretical and experimental resistivities for various metals as a function of film thickness**. The materials in these graphs are (**a**) aluminum, (**b**) copper, and (**c**) gold (the experimental data is adapted from Camacho and Oliva [14]).

When Equation 8 is graphed (resistivity vs. film thickness), the results are shown in Figures 3, 4, 5, and 6, and the theoretical data can easily be compared with the experimental data in the scientific literature [11-14]. The results show that a good match exists between the theoretical and experimental data, and therefore, the validity of Equation 8 is established. Furthermore, the experimental results include data from various materials (copper, aluminum, silver, and gold) as well as a data over a wide range of thicknesses; therefore, the general applicability of the theoretical model is also established.

In addition to matching theoretical data generated from the resistivity-film thickness equation with experimental data, a similar exercise can be performed with the resistivity-temperature equation (i.e., Equation 10). The experimental data available for this comparison involves thin films in which *t *> 2*l*_bulk_, so Equation 10 is used for establishing the relationship. This equation has been shown to provide an exact match with the Callendar-van Dusen equation, and thus, the theoretical model that produced this equation has been validated for bulk materials. By varying the value of *γ *in Equation 10, an exact match to the thin film experimental data should be obtained, and thus, the theoretical model will also be valid for metallic thin films.

**Figure 7:**
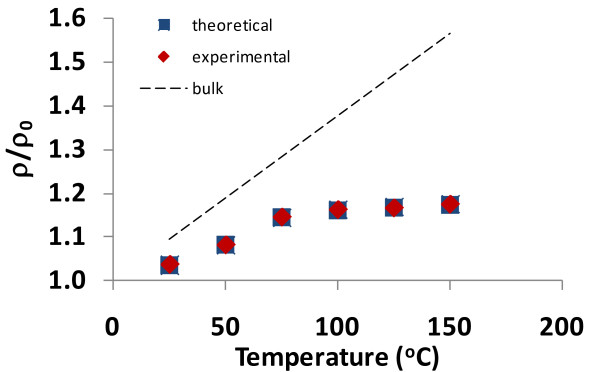
**Comparison of theoretical and experimental resistivities for a 46.3-nm-thick platinum film as function of temperature**. This graph represents all values of *τ*_1_/*τ*_2 _(the experimental data is adapted from Lacy [26]).

**Figure 8:**
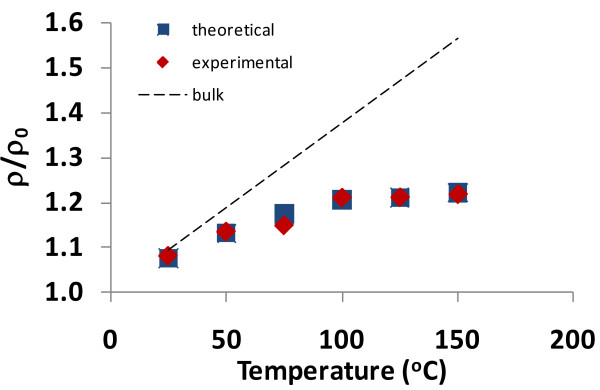
**Comparison of theoretical and experimental resistivities for a 74-nm-thick platinum film as function of temperature**. This graph represents all values of *τ*_1_/*τ*_2 _(the experimental data is adapted from Lacy [26]).

When Equation 10 is graphed (*ρ*/*ρ*_0 _vs. temperature) using parameters given in an unpublished study (F Lacy, unpublished work) and using *c *≅ 3.4, the results are shown in Figures 7 and 8. Figure 7 represents the relationship for a 46.3-nm platinum film, and Figure 8 represents the relationship for a 74.0-nm platinum film. In addition to the theoretical and experimental data shown in these graphs, a line is also provided to show how platinum in bulk form would respond. As shown in Figures 7 and 8, when the appropriate value for *γ *is selected, a perfect match between the experimental and theoretical values is obtained.

**Figure 9:**
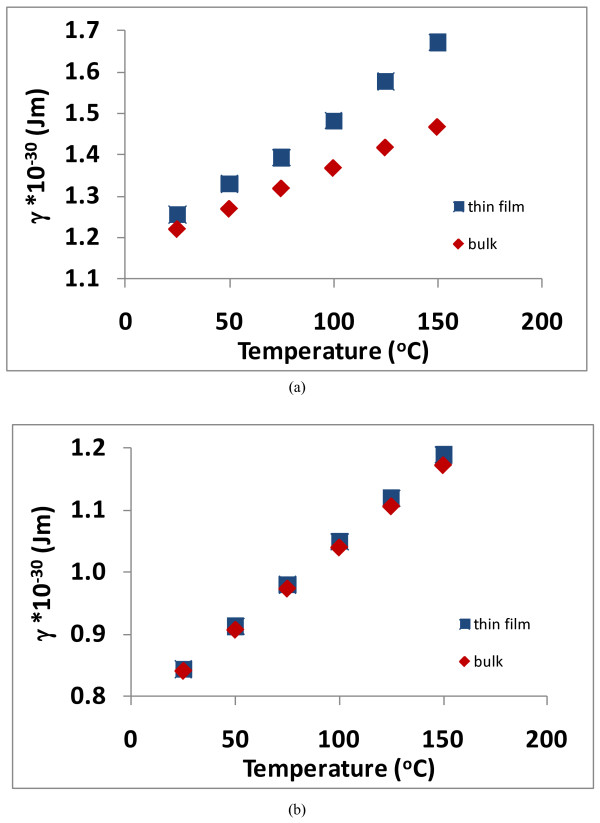
**Graph of *γ *vs. temperature for the 46.3-nm platinum thin film and for bulk platinum**. (**a**) *τ*_1_/*τ*_2 _= 5 and (**b**) *τ*_1_/*τ*_2 _= 50.

**Figure 10:**
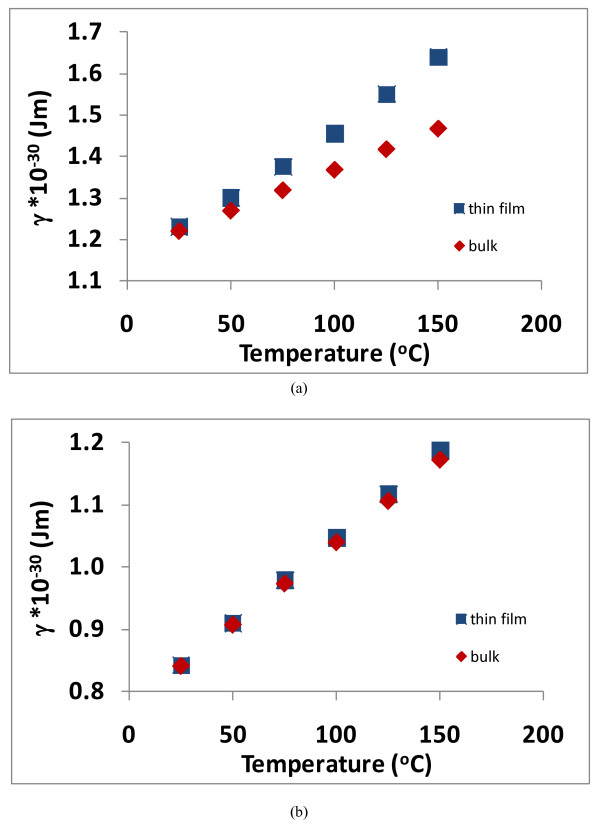
**Graph of *γ *vs. temperature for the 74-nm platinum thin film and for bulk platinum**. (**a**) *τ*_1_/*τ*_2 _= 5 and (**b**) *τ*_1_/*τ*_2 _= 50.

Additionally, graphs are also presented in Figures 9 and 10 showing how *γ *varies with temperature to produce the data shown in Figures 7 and 8. Depending upon the ratio of *τ*_1_/*τ*_2_, the values for *γ *will be different (F Lacy, unpublished work). When *τ*_1_/*τ*_2 _= 5, Figure 9a shows the values of *γ *for bulk platinum (which are the values that are used to produce the dotted line in Figure 7 as well as the Callendar-van Dusen coefficients for bulk platinum) and the values of *γ *for thin film platinum (with a thickness of 46.3 nm). It is seen from this figure that the values of *γ *for this thin film need to be larger than the bulk values to produce the thin film resistivity vs. temperature response. Figure 9b shows similar data when *τ*_1_/*τ*_2 _= 50, and it is seen that the values of *γ *for this thin film are only slightly larger than the corresponding bulk values.

Likewise when *τ*_1_/*τ*_2 _= 5, Figure 10a shows the values of *γ *that produce the bulk platinum resistivity vs. temperature response and the values of *γ *for thin film platinum (with a thickness of 74.0 nm). Also, just as in the case for the 46.3-nm film, Figure 10a, b shows that the values of *γ *for thin films needs to be larger than the bulk values to produce the thin film resistivity vs. temperature response, but the difference between bulk and thin film *γ *values are not as large for the 74.0-nm films as they are for the 46.3-nm film.

## Discussion

A theoretical model was developed to relate electrical resistivity to film thickness. Analysis of this model produced an equation for the electrical resistivity that is dependent upon the 'effective resistivity' when *t *≥ 2*l*_bulk_. Analysis of the model also produced an equation for the electrical resistivity that is dependent upon the effective resistivity as well as the effective thickness of the film when *t *≤ 2*l*_bulk_. To confirm or validate this theoretical model, experimental data were obtained from published research reports, and then the theoretical equation parameters were determined such that a good match between the theoretical and experimental data was achieved.

In performing this validation of the theoretical model, it is noted that this model is not an exact representation of a real conductor. It is an approximation, and with some estimations and assumptions, to make the analysis straightforward. It is also noted that collecting and recording experimental data is prone to error, so the recorded values in the literature will contain some inaccuracies. Furthermore, errors will also result in trying to determine the values of the experimental data by reading graphs from the research reports. In spite of all this, a very good match exists between the experimental data and values generated from the theoretical model, and thus, the theoretical model appears to be valid.

A theoretical model that develops the relationship between resistivity and temperature has been previously developed for bulk materials (F Lacy, unpublished work) [25]. However, in this paper, the primary equation from the aforementioned model was evaluated for its ability to characterize a thin film's response to temperature. It was determine that *γ *had to increase, compared with the bulk values, to reflect the change in the response of the atoms when the material has dimensions on the nanoscale. By adjusting the value of *γ *in the model and making them a little larger than the values used when the material is in bulk form, the theoretical values exactly matched the experimental data when the material is in thin film form. Additionally, in order to get the experimental and theoretical values to match, the value for *c *as used in Equation 10 had to be determined. The value of *c *was found to be approximately 3.4 (between 3.30 and 3.45), and it is noted that this is reasonable and also seen to be within the range of the values found in Table 1.

Additionally, it can be seen from the thin film temperature graphs in Figures 7 and 8 that the thin film resistivity response is closer to the bulk response for the 74.0-nm film than for the 46.3-nm film. This is expected because the 74.0-nm film is larger in size and thus 'closer' to bulk form than the 46.3-nm film. Also, Figures 9 and 10 show bulk and thin film values of *γ *as a function of temperature. By comparing Figures 9a and 10a, it can be seen that there is a smaller difference between the thin film and bulk *γ *values for the thinner film. The same assessment is true for Figures 9b and 10b, but the difference is less noticeable.

A simplified model and a straightforward equation are presented that relates electrical resistivity to film thickness and temperature. Although the temperature aspect is novel, there have been several papers that report on the changes in electrical resistivity of conductors due to scattering effects associated with thin films [6-22]. In general, these papers report on the use of complex models to analyze and understand the relationship between electrical resistivity and film thickness. The theoretical models in these papers either make assumptions which reduce the complexity (and make the equation easier to analyze), and as a result, the accuracy decreases, or these papers introduce additional variables (to account for specific scattering effects) to increase accuracy, but as a result, the complexity increases. The end result is that the models do not adequately explain the physics behind the results, or the equations cannot reproduce experimental results.

The model presented in this paper does not have the aforementioned limitations. It is not too complex nor is it too simple; it has a closed form solution that can be easily solved and accurately matches the experimental data. Since the model is founded on physics principles and since it is accurate, it provides valuable insight into the underlying physics that relates electrical resistivity to film thickness. Although the model groups some of the scattering effects together to keep it straightforward, the model can account for all of the different scattering effects (or stated another way, the model does not exclude any of the scattering effects).

Whenever a thin film or a nanofilm is fabricated in a laboratory, a perfect crystalline material will not be produced. An impure, polycrystalline material will typically be created. As a result of the fabrication process, lattice defects and impurities will occur throughout the material. The model presented in this paper lumps all of the effects associated with these imperfections and quantifies the effects as the effective resistivity associated with the material. This can be seen as a parameter that affects the material at larger thicknesses. The parameter or variable *c *used in the model indicates how close the material is to the ideal perfect crystalline structure. Materials where the value of *c *is closer to 1 represent a fabrication and processing method that produces a more ideal material.

Similarly, thin film fabrication can produce materials with grain boundaries and uneven surfaces, and these characteristics are known to affect the resistivity when the films are very thin. Obviously, the thinner the film is, the more dramatic these effects can be. Grain boundaries are prevalent and can be viewed as a means of reducing the electron mean free path in the same manner as a surface would. Furthermore, an uneven surface can be the result of a large uneven surface in one location, or it can be the result of many smaller uneven surfaces across the material. In either case, the model presented in this paper accounts for these effects using *t*' = *t *- *η*, which is the effective thickness. This variable can be understood as a parameter that affects the material at smaller thicknesses. Materials where the value of *η *is closer to zero represent a fabrication and processing method that produces a more ideal material.

Although the model presented in this paper cannot distinguish between some of the different scattering effects, for example, scattering from grain boundaries and from rough surfaces, it is very accurate in matching experimental data and thus will serve as a good tool to provide quick and accurate analysis of a material. (Note that grain boundary scattering is reported to be more dominant than scattering from rough surfaces and thus can be assumed to be the cause of scattering.) Since this model is very accurate and very easy to use, it could also be useful in performing initial analysis on a material to 'categorize' the effects, and then a more complex model could be used to help distinguish the different effects.

## Conclusions

In this paper, a two-dimensional theoretical model was created and developed. This model can be used to determine how the electrical resistivity of thin films is influenced by film thickness and temperature. The equation produced from this model was examined using experimental data, and it was found that the model generates accurate results. As a result, this model provides excellent insight into the underlying physical mechanisms by which the film thickness and temperature affect the electrical resistivity.

## Competing interests

The author declares that he have no competing interests.

## Authors' information

FL received his BSEE degree and Ph.D. degree in electrical engineering from Howard University, Washington, DC in 1987 and 1993, respectively, and his MSE degree in 1989, from Johns Hopkins University, Baltimore, MD, USA. He was a postdoctoral fellow in the Bioengineering Department, University of California, San Diego for 4 years, where he performed research in the area of biosensors. He was also with the US Food and Drug Administration, where he performed medical device reviews. In 2002, he joined the Electrical Engineering Department, Southern University and A&M College, Baton Rouge, LA, USA, where he is engaged in research and teaches courses in solid-state electronics, electrical and electronic circuits, and electronics-based sensors.
